# Thermo-sensitive hydrogels combined with decellularised matrix deliver bFGF for the functional recovery of rats after a spinal cord injury

**DOI:** 10.1038/srep38332

**Published:** 2016-12-06

**Authors:** He-Lin Xu, Fu-Rong Tian, Cui-Tao Lu, Jie Xu, Zi-Liang Fan, Jing-Jing Yang, Pian-Pian Chen, Ya-Dong Huang, Jian Xiao, Ying-Zheng Zhao

**Affiliations:** 1 School of Pharmaceutical Sciences, Wenzhou Medical University, Wenzhou City, Zhejiang Province 325035, China; 2 The Second Affiliated Hospital of Wenzhou Medical University, Wenzhou, 325000, China; 3 Biopharmaceutical R&D Center of Jinan University, Guangzhou, Guangdong 510000, China

## Abstract

Because of the short half-life, either systemic or local administration of bFGF shows significant drawbacks to spinal injury. In this study, an acellular spinal cord scaffold (ASC) was encapsulated in a thermo-sensitive hydrogel to overcome these limitations. The ASC was firstly prepared from the spinal cord of healthy rats and characterized by scanning electronic microscopy and immunohistochemical staining. bFGF could specifically complex with the ASC scaffold via electrostatic or receptor-mediated interactions. The bFGF-ASC complex was further encapsulated into a heparin modified poloxamer (HP) solution to prepare atemperature-sensitive hydrogel (bFGF-ASC-HP). bFGF release from the ASC-HP hydrogel was more slower than that from the bFGF-ASC complex alone. An *in vitro* cell survival study showed that the bFGF-ASC-HP hydrogel could more effectively promote the proliferation of PC12 cells than a bFGF solution, with an approximate 50% increase in the cell survival rate within 24 h (P < 0.05). Compared with the bFGF solution, bFGF-ASC-HP hydrogel displayed enhanced inhibition of glial scars and obviously improved the functional recovery of the SCI model rat through regeneration of nerve axons and the differentiation of the neural stem cells. In summary, an ASC-HP hydrogel might be a promising carrier to deliver bFGF to an injured spinal cord.

Traumatic spinal cord injury (SCI) is a devastating condition characterized by extensive tissue degeneration and severe neurological dysfunction, which affects 250,000 to 500,000 people worldwide^1^. In detail, 12,000 new cases of SCI occur every year in the USA, and the total number of American individuals living with SCI is estimated at ~259,000. The pathology of SCI has two stages. The primary injury is a mechanical impact afflicted directly on the spine. The secondary injury is a complex cascade of molecular events including disturbances in ionic homeostasis, local edema, ischaemia, focal haemorrhaging, oxidative stress and inflammation^2^, with apoptosis playing a vital role in the progressive degeneration of the injured spinal cord^3^,^4^. The failure of axonal regeneration in SCI partly results from a lack of neurotrophic factors, in addition to the expression of axonal growth-inhibiting molecules or inflammatory reactions. Specifically, bFGF is a fibroblast growth factor (FGFs) that regulates a variety of biological functions including neuroprotection, vasodilation, the stimulation of angiogenesis and the suppression of apoptosis^5^,^6^. However, either the systemic or local administration of bFGF solution has significant drawbacks because of the short half-life and the limited distribution to the injury site^7^. Therefore, new preparations of bFGF that can effectively deliver bFGF into the spinal cord and enhance its neural regeneration function are necessary.

An extracellular matrix is commonly used for the therapeutic reconstruction and repair of many tissues, including musculotendinous tissues^8^,^9^,^10^, the lower urinary tract^11^, the kidney^12^, the myocardium^13^,^14^, the oesophagus, the peripheral nerves^15^, and the central nervous system^16^,^17^, among others. Many recent studies have used the acellular spinal cord (ASC) extracellular matrix as a cell carrier to investigate its therapeutic effect on neural recovery^18^. ASC scaffolds show good biocompatibility with the host tissue^19^. Previous studies indicate that ASC scaffolds used for SCI regeneration have many beneficial intrinsic characteristics, such as nontoxic degradation products and a soft and flexible texture^20^. Additionally, ASC hydrogels also can provide the necessary scaffold to promote axonal recovery *in vivo*^21^. More importantly, because of the retained components of the native extracellular matrix, an acellular scaffold may mimic the native extracellular matrix to sequester growth factors and improve their stability, which may benefit promoting regeneration and functional recovery of injured neuron following SCI^22^. However, ASC is not complete compared to native spinal cord tissues because of damage to its fibre tract and a lack of a three-dimensional net structure after homogenization^23^.

Therefore, an optimized ASC is necessary for the reconstruction of the three-dimensional (3D) matrix structure and the controlled release of a sequestered growth factor. In our previous study, a temperature-sensitive heparin modified poloxamer (HP) polymer, which could spontaneously transfer from solution to hydrogel when temperature was increased to 35–38 °C was synthesized to control the release of encapsulated bFGF and achieved satisfactory nerve regeneration and the functional recovery of essentially paralyzed hind limbs in cases of SCI^24^. Additionally, HP hydrogels exhibited a 3D net structure and a good affinity and compatibility for biological tissues. An appropriately formulated HP hydrogel could encapsulate biological macromolecules and control the release of its contents *in situ*^25^,^26^. In the present study, an ASC was combined with an HP hydrogel to reconstruct a three-dimensional matrix structure. The neuroprotective factor bFGF was first inserted in an ASC scaffold to improve its stability, and then dispersed in a 3D HP hydrogel, resulting in a bFGF-ASC-HP hydrogel. We hypothesized that the ASC-HP hydrogel would facilitate neuroprotection in SCI via bFGF by controlling the release and diffusion of the bFGF in the injured spinal cord, realizing optimal neuroprotection in hemisected SCI model rats.

## Materials and Methods

### Reagents and Antibodies

All reagents used in this study were commercially available. Basic fibroblast growth factor (bFGF) was ordered from Gelusite Biology Technology Company, Zhejiang, China. Poloxamer 407-grafted heparin copolymer was synthesized by our laboratory as previously reported^27^. Dulbecco’s modified Eagle’s medium (DMEM) and fetal bovine serum (FBS) were purchased from Invitrogen (Carlsbad, CA, USA). Collagen types IV, laminin (LM), bFGF-R, fibronectin (FN), anti-GFAP, anti-GAP43, anti-Nestin and caspase-3 were purchased from Abcam (Abcam, CB, UK). Fluorescein isothiocyanate (FITC) was ordered from Sigma (Sigma-Aldrich, St. Louis, MO, USA). Cell Counting KIT-8 was purchased from Japan (CCK-8, Dojindo Laboratories Inc., Kumamoto, Japan).

### Animal and cells

Healthy adult female Sprague–Dawley rats about eight weeks (220–250 g) were purchased from Slac Laboratory Animal Corporation, Shanghai, China and housed at 23 ± 2 °C and humidity of 50 ± 10% controlled with a 12 h light/12 h dark cycle. Male rat adrenal pheochromocytoma, PC12 cells were purchased from the Cell Storage Center of Wuhan University (Wuhan, China).

### Preparation of acellular spinal cord

Donor rats used in the experiment were about eight weeks (220–250 g). Spinal cord was harvested under aseptic conditions. Spinal cord tissues were cut into 15 mm segments. Spinal cord was kept quickly into normal saline at the refrigerator (12 h, 4 °C). Then it was rinsed in 0.01% phosphate-buffered saline on gyratory shaker (1 h, 4 °C, 60 rpm) (PBS, Solarbio, Beijing). The spinal cord was rocked in l% of Triton X-100 solution (12 h, 4 °C, 120 rpm). Then the reagent was replaced with sterile distilled water and washed three times (each 10 min, 60 rpm). After that, 4.0% deoxycholate washed the sample (8 h, 4 °C, 120 rpm). At last, sample was eluted with 0.01% PBS ten times (10 min, 4 °C, 60 rpm). All solutions was contained with 200 U/ml penicillin (Invitrogen, 15140-122), 200 mg/ml streptomycin (Invitrogen, 15140-122). The sample in 0.01% sterile PBS could not be stored more than one week at 4 °C.

### HE staining and DAPI staining of acellular spinal cord

For histological and immunohistochemical analysis, the ASC was fixed with 4% paraformaldehyde in 0.01 M phosphate buffered saline (PBS, PH = 7.4) and dehydrated in enthanol series, then embedded in paraffin. Sections of 5 μm thickness were stained with haematoxylin and eosin (HE), sealed with neutrality gum and observed under a light microscope. Additionally, 4, 6-diamidino-2-phenylindole (DAPI), a fluorescent nucleic acid stain (VECTASHIELD mounting medium with DAPI, with an excitation wavelength of 350 nm and an emission wavelength of 460 nm) was also used to evaluate the remaining cells after decellularization for 5 min at 37 °C in the dark. All stained sections were analysed by fluorescence microscopy.

### Immunohistochemical staining of acellular spinal cord

Paraffin sections of 5 μm ASC were immunostained for collagen types IV, laminin (LM), and fibronectin (FN). The primary antibodies used were anti-mouse collagen type IV, (Abcam; 1:200), anti-mouse fibronectin (Abcam; 1:250), anti-mouse laminin (Abcam; 1:250) and anti-mouse bFGF-R (Abcam; 1:500) at 4 °C overnight. After extensive washing, the sections were incubated in the appropriate secondary antibodies (Molecular Probes, 1:100) for 1 h at 37 °C. ASC sections of bFGF-R were incubated with horseradish peroxidase-conjugated secondary antibodies for 2 h at 37 °C. Negative controls were similarly stained, but the primary antibodies were replaced with PBS.

### Glycosaminoglycan analysis of acellular spinal cord

Periodic acid-Schiff staining was performed on rehydrated paraffin sections of ASC to evaluate the retained glycosaminoglycans (GAGs). The staining procedure was carried out by placing the sections in an alcian blue solution for 15 min, a periodic acid solution for 15 min and a Schiff reagent for 20 min. The sections were washed well with tap water and placed in acid alcohol twice for 5 min each time. All of the slices were mounted in a non-fluorescent resinous medium.

### Affinity of ASC for growth factors

The ASC scaffold (50 mg) was soaked in 200 μL of a FITC-bFGF (100 μg/ml) solution at 4 °C for 24 h. The FITC-bFGF-ASC scaffold was removed into 50 mL of 0.1 M PBS, shaken continuously in a shaking incubator for 2 h, and then washed twice with fresh PBS. The FITC-bFGF-ASC was sectioned and analysed by fluorescence microscopy.

### Improving the stability of growth factors by ASC

Circular dichroism (CD) spectroscopy is a well-established technique for the study of proteins^28^. The effect of ASC on the stability of bFGF was explored by CD spectroscopy. The homogenized ASC solution was added into a bFGF solution and the mixture was gently stirred at 4 °C for 12 h. The final concentrations of ASC and bFGF in this ASC-bFGF mixture were 0.1 mg/ml and 0.3 mg/ml, respectively. To study the stability of bFGF at different temperatures, the ASC-bFGF solution was allowed to equilibrate at each temperature for 40–60 min before the CD spectrum was scanned. The same concentration of bFGF solution (0.1 mg/ml) in pH 7.4 PBS was used as the control. PBS/PBS containing ASC were used as baseline corrections before the CD scan. Two different test temperatures (4 °C and 55 °C) were set and the CD spectra was obtained using a MOS-450 spectrometer with a scanning range of 190 nm to 250 nm.

### Preparation and characterization of bFGF-ASC-HP hydrogels

Heparin-poloxamer (HP) was prepared according to the EDC/NHS method as previously described^29^. The bFGF-ASC-HP hydrogels were prepared using the cold method^30^. Briefly, ASC was homogenized into a suspension in pH 7.4 PBS and the ASC suspension was further lyophilized. The lyophilized ASC powder was added to the bFGF solution and incubated for 24 h at 4 °C. Finally, the HP powder was dissolved in the bFGF-ASC solution and then mixed at 4 °C with gentle stirring overnight. In the bFGF-ASC-HP mixture, the final concentrations of bFGF, ASC and HP polymer was 3 mg/ml, 10 mg/ml and 160 mg/ml, respectively. Except for the replacement of ASC with HP, a bFGF-HP solution with the same concentration of bFGF was also prepared by a similar process. To trace the distribution and *in vitro* release of bFGF from the hydrogel, bFGF was labelled with fluorescein isothiocyanate (FITC) by the method from the literature^27^. The bFGF was replaced with FITC-labelled bFGF (FITC-bFGF) to prepare a FITC-bFGF-ASC-HP solution for an *in vitro* release study.

### Gelation temperature measurement

The transition temperature was measured according to a method reported in the literature with some modifications^31^. Briefly, 5 ml of a bFGF-ASC-HP solution or an HP solution alone prepared as outlined above in a transparent vial was placed in a water bath and heated at a rate of 0.5 °C min^−1^ (from 22 °C up to 65 °C). Gel formation was indicated by a lack of movement of the meniscus when the vial was tilted; the temperature at which the immobility of the meniscus in each vial occurred was recorded as the sol–gel transition temperature. The apparent viscosity was also tested in a coaxial cylinder rheometer (DV-III, Brookfield, U.S.) with a SC4-14 rotor and a small sample adapter to determine if the hydrogel had a suitable viscosity for *in vivo* application. The apparent viscosity of the bFGF-ASC-HP solution or the HP solution alone at different temperatures was also measured, and viscosity curves were plotted against temperature.

### Surface morphology of ASC and bFGF-ASC-HP hydrogel

The lyophilized ASC samples were wiped on a copper sheet, and immediately immersed into liquid nitrogen. The samples were dried in a vacuum freeze-dryer for 24 h. The dehydrated specimens were cross-sectioned and sputter-coated with gold, and their surface morphology was observed by scanning electron microscopy (SEM) (Hitachi H-7500, Japan). The lyophilized bFGF-ASC-HP hydrogel samples were directly observed by SEM.

### *In vitro* release of bFGF from bFGF-ASC-HP hydrogel

To detect the bFGF release from temperature-sensitive bFGF-ASC-HP, FITC labelled bFGF was encapsulated into ASC-HP, to prepare a FITC-bFGF-ASC-HP hydrogel. The method for the *in vitro* release of FITC-bFGF was according to the literature^32^,^33^, with some modifications. Briefly, 1 mL of a FITC-bFGF-ASC-HP solution was placed in a tubes and allowed to gel completely at 37 °C. Then, 300 μl of pH 7.4 PBS was added to the hydrogel sample and the tube containing the hydrogel was placed in a shaking incubator at 37 °C and continuously shaken at a speed of 80 rpm/min. At predetermined time intervals, 300 μl of the release medium was sampled and replaced by an equal volume of fresh medium to maintain a constant volume. The fluorescence intensity of the as-prepared FITC-bFGF (*I*_*total*_) in the same volume of release medium was determined as the total drug amount, and the fluorescence intensity of the sample at different times (*I*_*t*_) was quantified with a Thermo Scientific Microplate Reader at λ_ex_ = 495 nm and λ_em_ = 525 nm. The cumulative release rate of the FITC-bFGF from the FITC-bFGF-ASC-HP hydrogel *in vitro* was calculated according to the following formula:


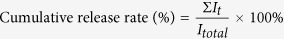


### *In vitro* cell proliferation assay

PC12 cells were used for the *in vitro* studies. The PC12 cells were cultured in DMEM high-glucose medium with 10% FBS and 1% penicillin and streptomycin in a humidified incubator (Thermo) under 5% CO_2_ at 37 °C. The cells were harvested in the logarithmic growth phase with trypsin for further experiments.

The cytotoxicity and proliferation of ASC-HP in PC12 cells were measured with a Cell Counting KIT-8. The PC12 cells were cultured in a 96-well plate at a density of 5,000 cells per well for 24 hours. Afterwards, the supernatant culture medium was removed and replaced with fresh medium (control). The ASC-HP hydrogel, the bFGF solution and the bFGF–ASC-HP hydrogel formed the various treatment groups. The concentration of bFGF in the culture medium was set at 40 ng/ml^34^. A 1.0 mg/ml bFGF solution was diluted to 800 ng/ml solution and used for the preparation of the bFGF solution and the bFGF-ASC–HP hydrogels. An aliquot of 10 μl of 800 ng/ml bFGF/bFGF-ASC–HP hydrogel was added into the medium (190 μl) to achieve a final concentration of bFGF of 40 ng/ml. After 24 h of treatment, the medium was replaced with flesh medium and microscopic graphs of the cell were recorded. An aliquot of 10 μL of the CCK-8 solution was added into a well and incubated for 2 h to quantify the cell proliferation. The absorbance was measured at 450 nm at a reference wavelength of 650 nm.

### Animal model of SCI and group of drug administration

All the animals were anaesthetized by an intraperitoneal injection of pentobarbital sodium (60 mg/kg, intraperitoneal), then placed on a constant temperature heating platform. The backs of the rats were shaved and the muscles above the midline were gently removed at the thoracic level. A laminectomy was performed on the thoracic vertebrae 9–10 (T9–T10). An incision was made with a fine scalpel blade through the meningeal membranes. Then, a hemisection was introduced at the right side of the spinal cord, sparing the left section. Animals in the sham group received the same surgical procedures without the resection. All of the animals were randomly divided into different groups. A dose of 20 μl (3 μg/μl) of bFGF solution/bFGF-HP hydrogel/bFGF-ASC-HP hydrogel was injected into the lesion using a micro syringe after the SCI. The rats in the sham group and the SCI group received the same dose of saline. After surgery, rats were returned and received manual bladder expression twice daily until bladder function was restored.

### Evaluation of the effects of bFGF treatment on behavioural function of SCI rats

The Basso, Beattie, and Bresnahan (BBB) scoring method was used to assess the rats’ open field locomotor function at 0 d, 3 d, 7 d, 14 d and 28 d. Two trained investigators who were blind to the experimental conditions scored the locomotion recovery in an open field according to the BBB scale. The BBB scale ranges from 0 (indicating no observed hind limb movement) to 21 (representing a normally ambulating rodent). The rats were placed individually on open fields and allowed to move freely for 5 min. All of the rats were evaluated every 2 days from day 0 to day 14 following the surgical procedure.

A footprint analysis was performed by dipping the animal’s hindpaws in dye^35^. All of the rats were allowed to walk across a narrow box (1 m long and 7 cm wide). The footprints were scanned and the digitized images were analysed.

### Regeneration of the injured spinal cord and the neuroprotective effect of the bFGF-ASC-HP hydrogel

#### HE staining of spinal cord

After 28 days, the rats were anesthetized by intraperitoneal injection with 1% pentobarbital sodium (60 mg/kg) and perfused with 0.9% NaCl, followed by 4% paraformaldehyde in 0.01 M PBS (pH = 7.4). The spinal cords from the T_8_–T_10_ level were excised and stored in cold 4% paraformaldehyde overnight, then embedded in paraffin. Longitudinal sections (5 mm thick) of the embedded spinal cord were mounted on poly-L-lysine-coated slides and stained with haematoxylin and eosin (HE) before observation under the light microscope.

#### Immunohistochemical staining

Immunohistochemical staining was applied to detect the protein expression in each experimental group. The 5 μm thick paraffin sections of the spinal cords were incubated at 4 °C overnight with the primary antibodies anti-Nestin (Abcam; 1:500), anti-GAP43 (Abcam; 1:500), anti-Neuron (Abcam; 1:500) and anti-Caspase 3 (Abcam; 1:500), then washed with PBS three times. The paraffin sections were incubated with horseradish peroxidase-conjugated secondary antibodies for 2 h at 37 °C. The reaction was stopped with 3, 3-diaminobenzidine (DAB). The results were imaged using an optical microscope (Nikon ECLIPSE Ti-S, Ruikezhongyi Company, Beijing, China). The total number of immunoreactive cells in each representative mesencephalic section was counted for the striatum region by technicians who were blind to the treatment. The quantification of each density was performed using the Image-Pro Plus software.

#### Apoptosis assay

DNA fragmentation *in vivo* was detected using a one-step TUNEL Apoptosis Assay KIT (Roche, Mannheim, Germany). The images were captured with a Nikon ECLIPSE Ti microscope (Nikon, Japan).

### Statistical Analysis

The data were expressed as the mean ± standard error of the mean (SEM) of three independent experiments. The statistical significance was determined with Student’s t test for two experimental groups. If more than two groups were compared, the statistical evaluation of the data was performed using a one-way analysis of variance (ANOVA) and the Newman–Keuls test. *P* values < 0.05 were considered statistically significant.

### Ethics Statement

This study was performed in strict accordance with the recommendations in the Guide for the Care and Use of Laboratory Animals of the National Institutes of Health. All animal experiments were performed with the approval and according to the guidance of the Institutional Animal Care and Use Committee of Wenzhou Medical University (Permit Number: XI104079).

## Results

### Preparation and characterization of ASC

**Figure 1:**
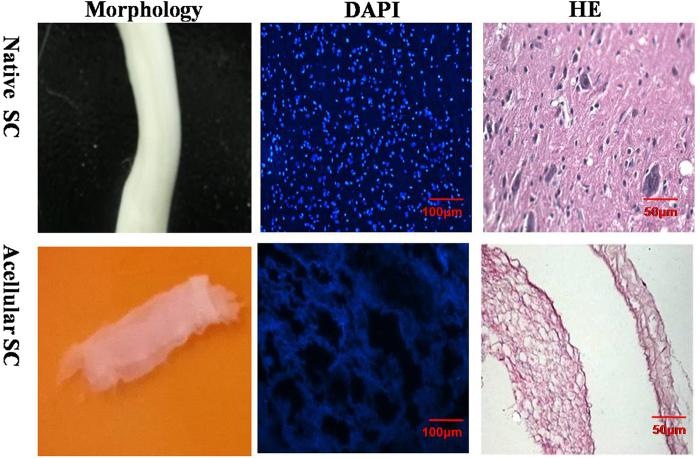
Decellularization of spinal cord. (**A**) The morphology of native spinal cord and ASC; (**B**) The DAPI staining of native spinal cord and ASC(×200). Scale bar, 100 μm; (**C**) :The H&E staining of native spinal cord and ASC (×400, scale bar, 50 μm, random sampling, n = 3).

Figure 1 shows that ASC had a transparent architecture, the bulk shape of which was similar to the native spinal cord. DAPI and H&E staining did not reveal any cellular components in the ASC scaffold, but the native spinal cord had a large amount of integrated nuclei in the spinal cord cells (Fig. 1). These results suggested that the spinal cord was completely decellularised by the 1% Triton X-100 treatment, which was consistent with the literature.

**Figure 2:**
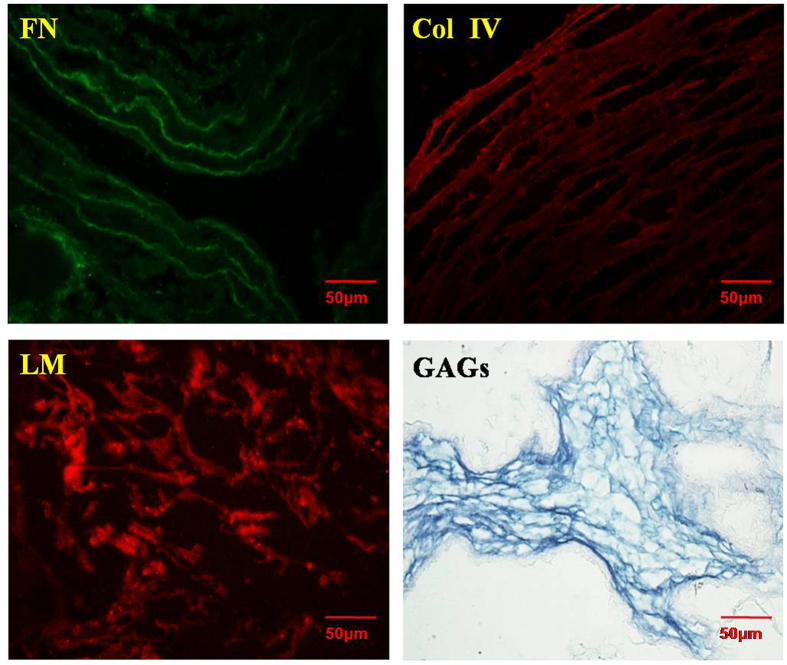
The components of acellular spinal cord. The preservation of collagen IV, laminin, fibronectin in ASC by immunofluorescence staining; Magnification was ×400. The PAS staining of ASC (×400, scale bar, 50 μm, random sampling, n = 3).

To confirm whether components related to the extracellular matrix were preserved in the ASC scaffold, the ASC was stained by immunofluorescence staining to evaluate the fibronectin (FN), collagen IV (Col IV), and laminin (LM) contents. The results are shown in Fig. 2. All of these components were obviously positively stained, indicating that the extracellular matrix-related proteins were preserved following decellularization. Additionally, polysaccharide components such as glycosaminoglycans (GAGs) were also evaluated by periodic Acid-Schiff staining. Positive staining of GAGs was observed, suggesting that polysaccharide components were also retained in the ASC scaffold. The preservation of extracellular matrix proteins and polysaccharides was very important to sequester the growth factor and stabilize it.

### Affinity of ASC for growth factors

**Figure 3:**
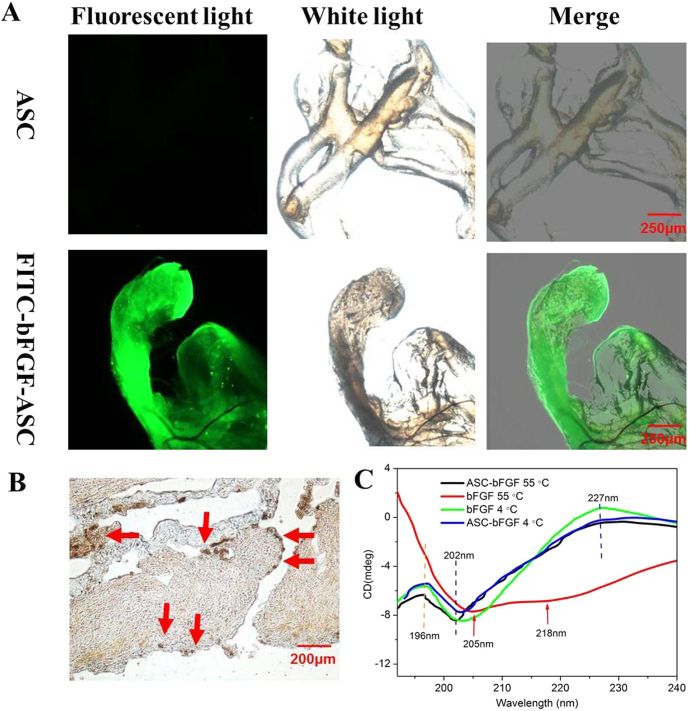
Affinity of ASC for growth factors. (**A**) Microscopic images of ASC scaffold and ASC scaffold incubated with FITC-bFGF was placed in under the fluorescence light and white light (×40, scale bar, 250 μm). (**B**) The bFGF-R of ASC scaffold by the immunohistochemical staining. Magnification was ×400. Scale bar, 50 μm. (**C**) CD spectra of bFGF solution and bFGF binding with ASC after 1 h of exposure to different temperature. The concentration of bFGF is 0.1 mg/ml. The concentration of ASC was 1 mg/ml. (Random sampling, n = 3).

Sulphated glycosaminoglycan (GAGs) are known for its affinity for a variety of growth factors and their ability to sequester those growth factors in the extracellular matrix. To investigate whether the affinity of the ASC scaffold for the growth factor was compromised, the ASC scaffold was incubated with FITC-bFGF and then washed twice under harsh conditions. A fluorescence image of the ASC scaffold was subsequently observed by fluorescence microscopy. The results are displayed in Fig. 3. The blank ASC scaffold showed no fluorescence, but the strong fluorescence of FITC-bFGF was distributed along the ASC scaffold despite the washings with fresh medium, indicating its strong affinity for bFGF. Except for electrostatic attractions between the N- and O-sulphated residues of the sulphated GAGs and the arginine and lysine residues in bFGF, receptor-ligand shape recognition might have also made a tremendous contribution to the strong affinity between the ASC and the bFGF. The existence of bFGF receptors was confirmed by immunohistochemical staining using its antibody, and the results are shown in Fig. 3B. Numerous bFGF receptors (brown-staining dots) were dispersed on the ASC scaffold. Although the bFGF receptors that initiated a biological effect was mainly localized on the surface of active cells, several studies have also detected high affinity receptors for FGFs in the extracellular matrix^36^,^37^,^38^. Similar results in this study further verified the receptor-ligand shape recognition between ASC and bFGF.

### ASC improves the stability of bFGF

Human bFGF naturally exists in a coil-like conformation or distorted antiparallel β-sheets or in both forms. Its conformation was irreversibly converted to a helix-like conformation upon heating the solution to above 55 °C, and the presence of that form could be easily monitored by the CD spectrum. After exposure to various temperatures for 1 h, the CD spectra of bFGF was scanned to investigate its stability. The results are shown in Fig. 3C. The CD spectrum of a free bFGF solution at 4 °C showed a group of typical peaks at 196 nm (positive), 202 nm (negative) and 227 nm (positive), indicating the existence of the coil-like and β-sheet conformations^39^, while its conformation transitioned to the α-helix conformation with a double minimum at 205 and 218 nm after 1 h of exposure to 55 °C, indicating the instability of the bFGF solution with increasing temperature. Interestingly, the binding of bFGF to ASC was still stable even when the temperature was increased to 55 °C, showing no obvious peak shift in the CD spectrum. These results suggested that ASC also improved the stability of bFGF, possibly due to the high affinity of ASC for bFGF, as tested above.

### Characterization of the bFGF-ASC-HP hydrogel

**Figure 4:**
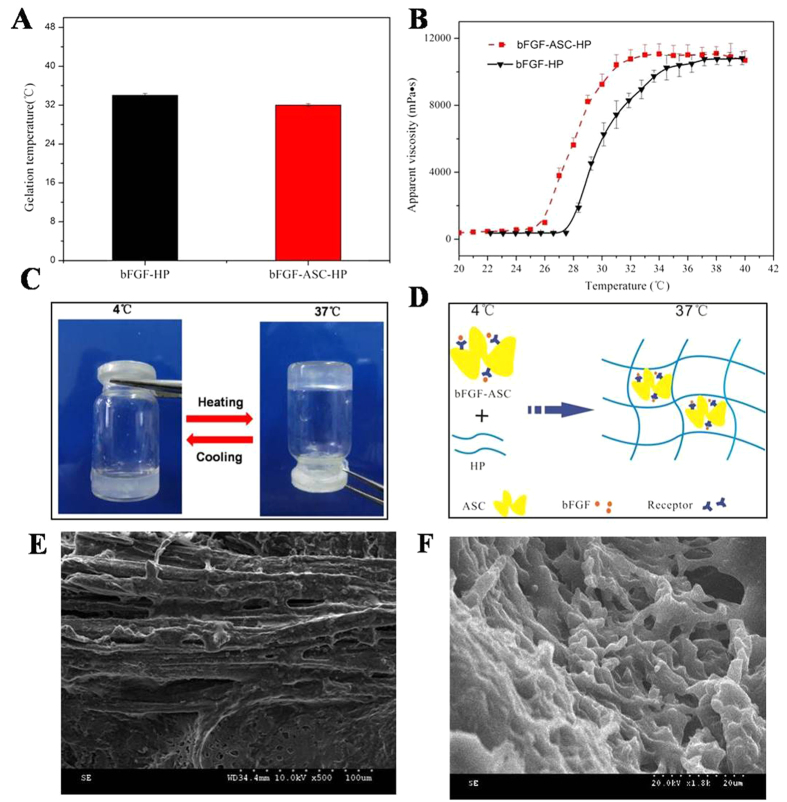
Characteristics of ASC and bFGF-ASC-HP hydrogel. (**A**) Gelation temperatures were measured using a rheometer. (**B**) Determination of apparent viscosity. The concentration of HP was 16% (w/w). (**C**) The state of bFGF-ASC-HP hydrogel at different temperatures (4 °C and 37 °C). (**D**) Schematic diagram of the state bFGF-ASC-HP hydrogel under different temperatures (4 °C and 37 °C). (**E**) SEM image of the surface morphology of ASC (×1000). (**E**) SEM image of the morphology bFGF-ASC-HP hydrogel (×1800). Random sampling, n = 3.

Our previous study indicated that 16% (w/w) of the HP solution quickly transitioned to a gel state as the temperature increased. To investigate whether the bFGF-ASC complex compromised the gelation of HP, the gelation temperature of a bFGF-ASC-HP solution and its apparent viscosity as a function of temperature was determined. The gelation of the HP solution alone occurred at approximately 25 °C and quickly transitioned to the hydrogel state within 100 seconds as temperature was increased to 34 °C. The addition of the bFGF-ASC complex had no significant effect on the gelation properties of the HP solution or the gelation temperature of the bFGF-ASC-HP solution at 32 °C. According to results in the literature^40^, the ideal apparent viscosity of a hydrogel for *in vivo* application was approximately 10000 mPa s. The apparent viscosity of the 16% HP hydrogel increased from 8000 mPa s to 10000 mPa s as the temperature was changed from 32 °C to 37 °C and reached a stable plateau at 10000 mPa s, without a further increase of temperature (Fig. 4B). Additionally, the viscosity curve of the bFGF-ASC-HP solution has similar rheological properties as the HP solution alone, with a stable apparent viscosity of 10000 mPa s.

To reconstruct the 3D network structures, the bFGF-ASC complex was subsequently added to an HP solution to prepare a suspension of bFGF-ASC-HP at a lower temperature (4 °C). As the temperature increased to 37 °C, the liquid suspension of bFGF-ASC-HP had gradually transitioned to a hydrogel state within 100 seconds, indicating that the addition of the bFGF-ASC complex did not compromise the temperature-sensitivity of the HP polymer. The transition from a solution to a gel embedded the bFGF-ASC platform in the 3D hydrogel network, as shown in Fig. 4D, which was confirmed by SEM, and those results are shown in Fig. 4E and F. The SEM micrographs of ASC showed the fibrous architecture with inner pores on the surface, while the lyophilized bFGF-ASC-HP hydrogel powder had a 3D network floccule that embedded the fibrous ASC. Additionally, the porous structure of the bFGF-ASC-HP hydrogel powder was also observed in the SEM image, which was favoured the release of bFGF from the hydrogel.

### *In vitro* release of bFGF from bFGF-ASC-HP hydrogels

**Figure 5:**
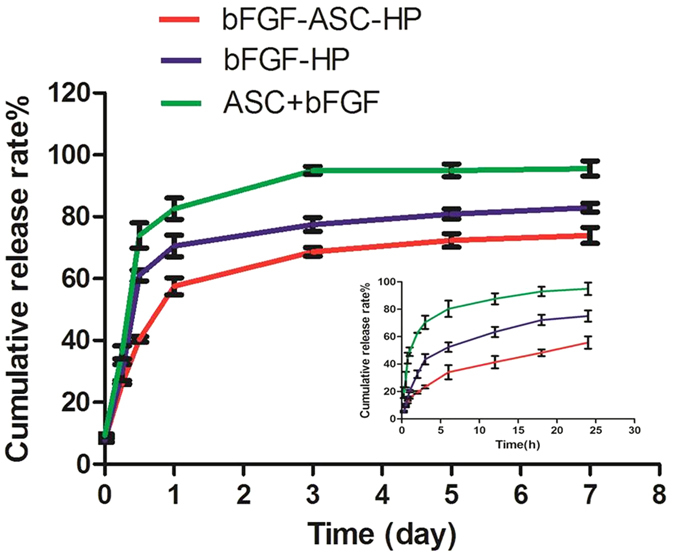
The cumulative release of bFGF *in vitro*. Different time points are 15 min, 30 min, 45 min, 1 h, 3 h, 6 h, 12 h, 18 h, 24 h, 3 d, 5 d and 7 d. Data are presented as Mean ± SEM, n = 3.

The embedding of the bFGF-ASC complex in a hydrogel network structure was favourable to sustain the release of the drug. The results of the *in vitro* release of the bFGF from the bFGF-ASC-HP hydrogel, the bFGF-HP hydrogel and the bFGF-ASC complex are shown in Fig. 5. Because of the lack of a 3D network architecture in the ASC scaffold, the bFGF-ASC complex released the bFGF rapidly. The cumulative release of bFGF was as high as 70% within 4 h. In contrast, a slower release of bFGF from the HP hydrogel matrix was observed whether bFGF was combined with ASC or not. However, when bFGF was first combined with the ASC scaffold before addition to the hydrogel, a slower release of bFGF from the bFGF-ASC-HP hydrogel was observed, comparing to release from the bFGF-HP hydrogel. The respective cumulative release percentages at 4 h and 24 h for the bFGF-HP hydrogel were 42% and 65%, while the respective percentages of cumulative release at 4 h and 24 h for the bFGF-ASC-HP hydrogel were only 21% and 42%. This indicated that the receptor-mediated interaction between bFGF and ASC, which was previously demonstrated, could block the release of bFGF from the ASC. A determination of the cumulative release for 7 days revealed that the full release of bFGF in ASC occurred on the third day, but the bFGF-HP and bFGF-ASC-HP hydrogels required 7 days for a complete release and had no obvious difference on the release of the late.

Because most of bFGF may bind with heparin block of heparin modified poloxamer (HP) polymer in hydrogel-based matrix, an initial burst release of ca.57% within the first day was observed. However, there exhibited a very slow release, and about 1% of the encapsulated bFGF per day was released over 3–7 days (Table S1), this was due to the strong affinity of parts of bFGF with ASC matrix HP hydrogel. After 7 day of release, it was interfered that about 25% of the encapsulated FITC-bFGF was retained in hydrogel for bFGF-ASC-HP. To confirm if the remaining bFGF in hydrogel was still stable, the remaining bFGF in different formulations was further determined by ELISA and SDS-PAGE electrophoresis after 7 day of release. As shown in Table S2, the amount of the remaining bFGF in bFGF-ASC-HP was determined to be 24.16 ± 0.25%. The result was identical to the interfered value of the remaining FITC-bFGF *in vitro* release profile, indicating the remaining bFGF is relatively stable. Also, the molecule weight of bFGF was not changed as exhibited by SDS-PAGE electrophoresis analysis (Fig. S3), further indicating the remaining bFGF was still stable.

### *In vitro* cell proliferation assay

**Figure 6:**
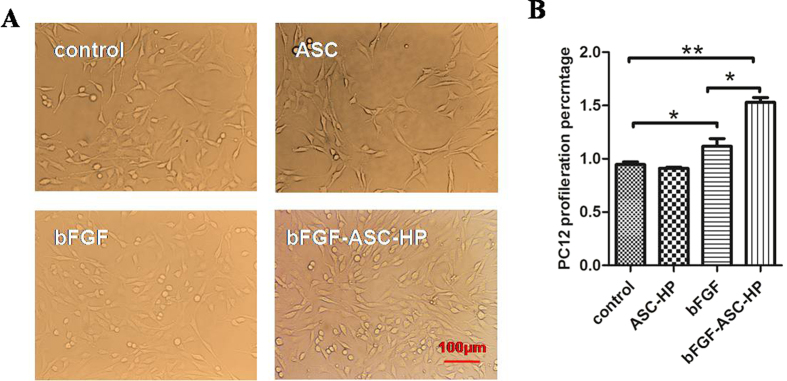
Toxicity assay of bFGF-ASC-HP hydrogel. (**A**) Microscopy showing growing state of PC12 cells between bFGF-ASC-HP hydrogel group and normal cells (×200, scale bar, 100 μm). (**B**) CCK8 assay of PC12 cell survival rate in various groups. Data are presented as Mean ± SEM, n = 5. *P < 0.05, **P < 0.01 and ***P < 0.01.

The pheochromocytoma-derived PC12 cell line, a neoplastic rat cell line arising from neural crest tissue^41^, was used as a model of neural-like cells to evaluate the *in vitro* cytotoxicity of the bFGF-ASC-HP hydrogel. Figure 6A shows microscopic images of PC12 cells after incubation with the various treatments for 24 h. No sign of a loss of cell adherence, nuclear condensation and cell soma contraction were noted, indicating that no treatments were cytotoxic. Conversely, significant proliferation of PC12 cells was noted after treatment with a bFGF solution or a bFGF-ASC-HP hydrogel. Compared to the control group, the PC12 cells proliferated by approximately 50% within 24 h after treatment with the bFGF-ASC-HP hydrogel (Fig. 6B), which was significantly higher than that for the bFGF solution. These results suggested the occurrence of a sustained-release of bFGF from the bFGF-ASC-HP hydrogel that might be beneficial for cell proliferation.

### Motor function

**Figure 7:**
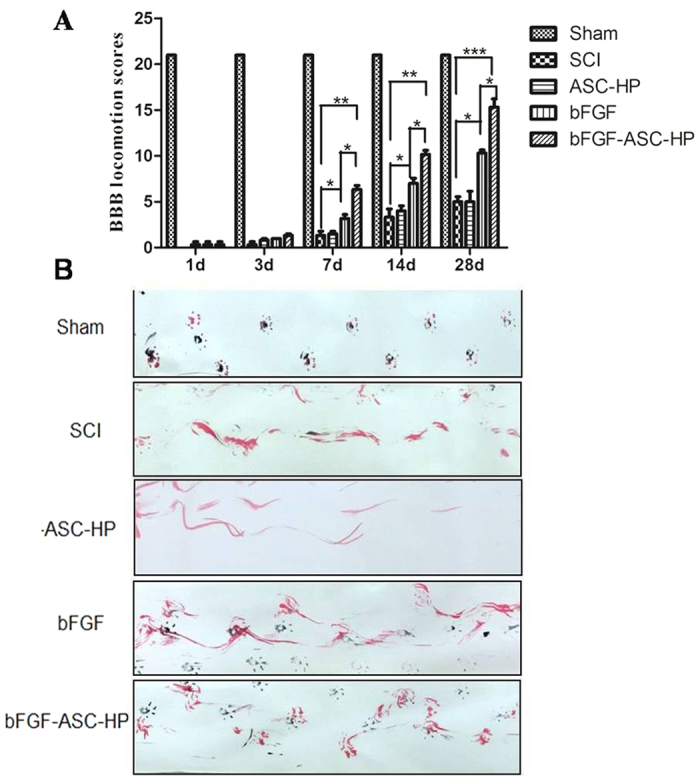
Motor function locomotion assessments of SCI rats at different time points in different groups. (**A**) BBB locomotion assessments of SCI rats. The BBB scores of the sham, SCI, ASC-HP, bFGF solution, bFGF-ASC-HP hydrogel group. The score of sham group was 21 points, which means normal locomotion. (**B**) Footprint analyses of the different groups. Data are presented as Mean ± SEM, n = 5. *P < 0.05, **P < 0.01 and ***P < 0.01.

The BBB rating scale and footprint recordings at 1, 3, 7, 14 and 28 days are usually used to evaluate the functional recovery from SCI in rats^42^. The sham group had a scores of 21. The BBB scores indicated that all SCI rats suffered from complete paralysis of the right hind limbs (BBB score = 0, at day 1 after SCI). One and 3 days after injury, no significant difference in the BBB scores between treated groups and the SCI group was evident. Fourteen days after injury, although the BBB scores of the ASC-HP hydrogel groups had not significantly increased compared to the SCI group, an obvious improvement of the BBB scores was apparent for both the \bFGF-ASC-HP hydrogel and bFGF solution groups, indicating that good recovery of locomotor function (*P* < 0.05; Fig. 7A) had occurred. At 7 d and 14 d after injury, the BBB scores for the bFGF-ASC-HP hydrogel group were significantly higher than that for the bFGF solution group (*P* < 0.01), suggesting a better recovery of locomotor function. Similar results were seen for the footprint analyses. Rats treated with the bFGF-ASC-HP hydrogel showed clear toe dragging and consistent hindlimb coordination. In contrast, the SCI group and the bFGF groups showed inconsistent coordination and extensive toe dragging as revealed by the ink streaks extending from both hindlimbs (Fig. 7B). These results indicated that bFGF-ASC-HP promoted locomotor recovery of SCI rats and might enhance the neuroprotective effect of bFGF for SCI.

### Regeneration of injured spinal cord and neuroprotective effect of bFGF-ASC-HP hydrogel

**Figure 8:**
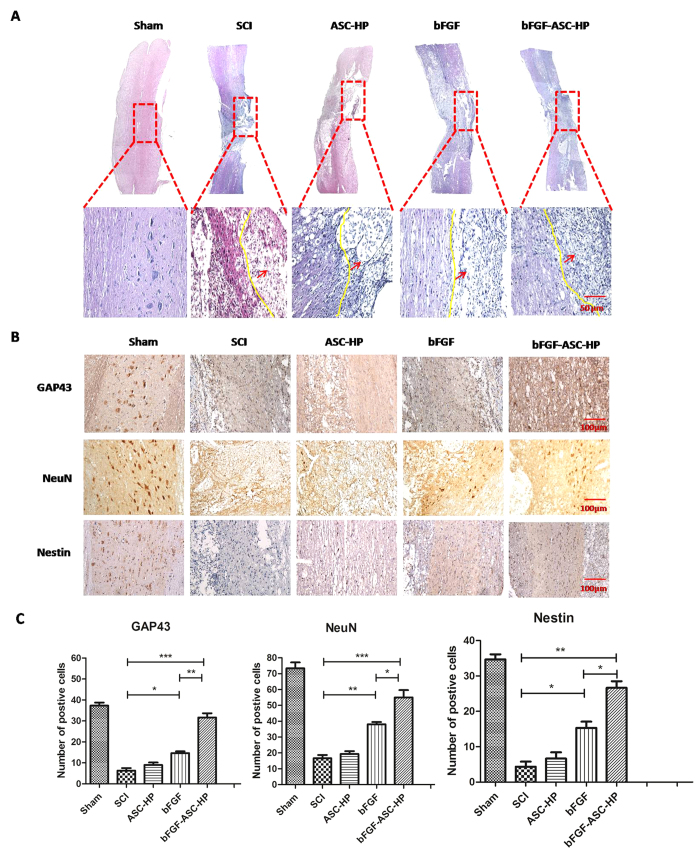
The neuroprotective effect of bFGF-ASC-HP hydrogel in SCI. (**A**) HE staining of tissue at the junction of the injured site and normal tissue (The red arrows point to the semi - cut damage area). The expression of GAP-43, neuron, nestin the immunohistochemical staining of crosscutting results on the 14th days by bFGF-ASC-HP hydrogel treatment after spinal cord injury; magnification was ×200. (**B**) Analysis of the positive cells of the immunohistochemistry results. Data are presented as Mean ± SEM, n = 5. *P < 0.05, **P < 0.01 and ***P < 0.01.

The HE staining results of spinal samples from the experimental groups after 28 days are shown in Fig. 8A. After SCI, the injured spinal cord had a large cavity around the injury site with a widespread loss of white matter and central grey matter. Although the ASC-HP material alone did not promote the repair of the injured spinal cord (a similar cavity and loss white matter and central grey matter was noted), these signs were significantly attenuated by the bFGF solution and the bFGF-ASC-HP, indicating that bFGF induced regeneration of the damaged spinal cord. Moreover, compared with the bFGF solution, the bFGF-ASC-HP hydrogel treatment group showed a more obvious therapeutic effect, without a cavity around the damaged site and a better regeneration of the white matter and the central grey matter. These results further strengthened the conclusion that transplantation with a bFGF-ASC-HP hydrogel fills the cavity at the damaged site and may have a neuroprotective effect.

To confirm the neuroprotective effect of bFGF-ASC-HP on rats with SCI, the level of GAP43, neuron and Nestin in SCI rats was determined after treatment by immunohistochemical staining, and the results are shown in Fig. 8. GAP43 is a known marker of an axon membrane protein and is involved in nerve cell growth, synapse formation and the regeneration of nerve cells^43^. Compared with the SCI group, SCI rats treated with the bFGF solution and the bFGF-ASC-HP hydrogel showed an increase in GAP43 positive cells (*P* < 0.05), and the expression of the GAP43 protein was significantly higher after treatment with the bFGF-ASC-HP hydrogel than with the bFGF solution (*P* < 0.01), indicating that the bFGF-ASC-HP hydrogel had an enhanced ability to induce axon regeneration (*P* < 0.01). After SCI, astrocytes excessively proliferate and form a glial scar surge, which resulted in an impedance of the functional recovery of SCI^44^. Compared with the sham group, the SCI group showed significant pathological changes. The survival rate of neurons in the SCI rats was decreased. The immunofluorescent staining of neuron-specific marker neuronal nuclei (NeuN) is shown in Fig. 8. The number of positive NeuN cells decreased significantly in 14 days in SCI rats and was higher in bFGF treated rats. However, the number of positive NeuN in the bFGF-ASC-HP hydrogel group was greater than that in the bFGF solution group (*P* < 0.01, Fig. 8C). Nestin, an intermediate filament protein, has been considered as a marker for precursor neural cells and neural stem cells in rat spinal cord^45^. A high expression of nestin protein was well-related to neuron repair. The level of nestin in SCI rats in the various treatment groups was also determined by immunohistochemical staining. Similarly, compared with the SCI group, nestin-positive cells was obviously increased after treatment with the bFGF solution or the bFGF-ASC-HP hydrogel (*P* < 0.05), indicating the activation of the neural stem cells pathway. Additionally, the level of nestin expression after treatment with the bFGF-ASC-HP hydrogel was significantly higher than that for the treatment with the bFGF solution (*P* < 0.05) (Fig. 8D). In summary, the therapeutic mechanism of bFGF-ASC-HP in the functional recovery after SCI was due to a synergistic effect of the facilitation of the regeneration of the neural axons, the inhibition of the proliferation of astrocytes and the activation of the neural stem cells pathway.

### Inhibition of Cell Apoptosis

**Figure 9:**
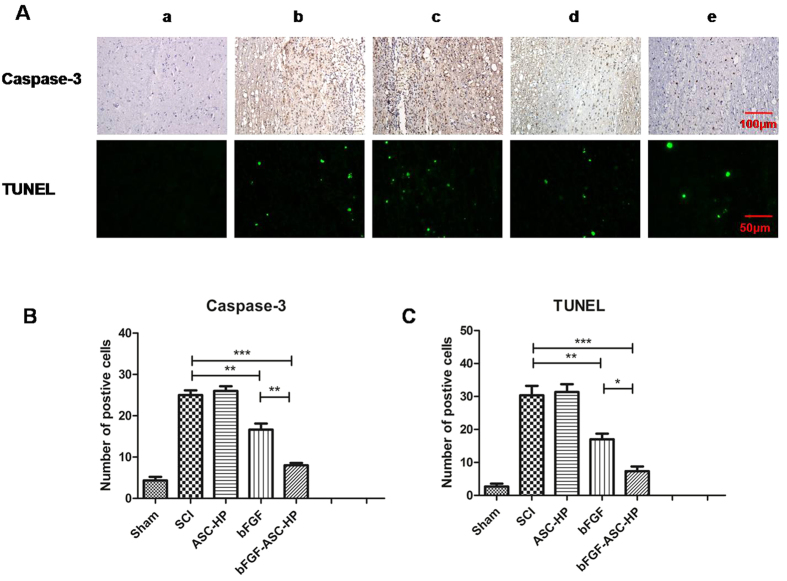
Apoptosis results of SCI rats after bFGF-ASC-HP hydrogel treatment. (**A**)The expression of Caspase-3 in the immunohistochemical staining (×200 scale bar, 100 μm) and immunofluorescence results of the TUNEL assay (×400, scale bar, 50 μm). a: Sham group; b: SCI group; c: ASC-HP hydrogel group; d: bFGF solution group; e: bFGF-ASC-HP hydrogel group. (**B**) Analysis of the positive cells of the immunohistochemistry results and the analysis of apoptosis cell by bFGF-ASC-HP hydrogel treatment after spinal cord injury. All experiments were performed in three repetitions. Data are presented as Mean ± SEM, n = 5. *P < 0.05, **P < 0.01 and ***P < 0.01.

The inhibition of apoptosis after treatment with the bFGF-ASC–HP hydrogel was also evaluated by caspase-3 staining and TUNEL staining. The results of the caspase-3 staining after treatment with the various formulas is shown in Fig. 9A. After SCI, the number of apoptotic cells significantly increased in the SCI group (Fig. 9A), while the caspase-3-positive cells at 14 d after treatment with the bFGF-ASC–HP hydrogel were significantly reduced to a level lower than that of the bFGF solution groups (*P* < 0.01) (Fig. 9A and B). Similar results were observed in the TUNEL staining analysis (Fig. 9A,C). The bright green dots were deemed as apoptosis-positive cells in the lesions. As shown in Fig. 8A, non-apoptotic cells occurred in the sham group. Significantly fewer apoptotic cells were found in the bFGF-ASC–HP hydrogel group than in the bFGF solution group (*P* < 0.05) and the SCI group (*P* < 0.001) (Fig. 9C). These results demonstrated that the inhibition of apoptosis might also contribute to the neuroprotective effect of the bFGF-ASC–HP hydrogel after SCI.

## Discussion

Spinal cord injury tends to result in the loss of neurons and the formation of cystic cavities, which inhibit axonal regeneration in the neural area with the lesion. Despite vast improvements in therapy after SCI, an optimum protocol has not been developed for clinical application because of a restricted number of therapeutic agents. Recently, because of their tuneable physical properties, *in situ*-formed hydrogels have been extensively studied for SCI regeneration^40^. An injectable hydrogel should be formulated to have mechanical properties closely matching the extracellular matrix of the native spinal cord. Additionally, an injectable hydrogel can accommodate cellular and molecular therapeutics to modulate the wound environment^46^ and enhance regeneration^40^. Hydrogels are a water-swollen insoluble polymer 3D network that have a wide range of chemical compositions and properties^47^,^48^,^49^. Conventional hydrogels are not suitable for encapsulating growth factors for SCI treatment because of their poor affinity for the growth factors and their poorly tuneable mechanical strength at normal body temperature. Additionally, conventional hydrogels do not provide enough nutrition for the growth of endogenous cells. In our previous studies, we prepared a novel heparin-poloxamer (HP) hydrogel that had a high affinity for NGF and a controlled phase transition temperature for *in situ* administration in SCI^50^. To mitigate the limitations of conventional hydrogels during SCI treatment, an HP hydrogel is usually combined with a biologically active substance, which exerts the therapeutic effect in SCI therapy. Many studies have suggested that a decellularised extracellular matrix had the potential to induce endogenous progenitors at a damaged CNS site, causing proliferation and the differentiation of functional cells to replace lost CNS tissue in cases of injury^51^,^52^,^53^,^54^. ASC is an extracellular matrix-based biomaterial that has many inherent advantages over synthetic materials for surgical and regenerative medicine applications. This protein-based material has significant mechanical strength, retains its biological activity with low immunogenicity and can promote tissue regeneration. More importantly, they could sequester growth factors in their cavity by a receptor-mediated affinity interaction and improve stability. In the present study, an injectable ASC-HP hydrogel encapsulated bFGF that combined the advantages of the unique properties of ASC and HP was developed to investigate its therapeutic efficacy after SCI. All of the results indicated a key role for bFGF-ASC-HP in promoting neural recovery in SCI.

First, an ASC scaffold was successfully prepared from the spinal cords of healthy rats. Figure 1 shows that the ASC was completely decellularised with no cell nuclei remaining. In addition to the bioactive molecules embedded in the ASC scaffold that might be released during the degradation of the scaffold, cryptic fragments such as collagen, fibronectin and laminin were created by cleavage of the parent molecules^54^,^55^,^56^. Those three proteins are the primary proteins of the ASC and are very important nutritional components that promote neural growth and provide mechanical protection. For example, laminins are axonal growth-promoting proteins and play a key role in guiding commissural axons to the midline^55^. Collagen fibres can provide the mechanical support for the combined hydrogel^50^,^57^. More importantly, GAGs are also preserved in the ASC and can absorb growth factors and cytokines to promote cell adhesion and migration.

In our previous study^50^, a HP hydrogel assumed a liquid state at room temperature, and it could transition to a three-dimensional network structure at normal body temperature. In the present study, the bFGF-ASC-HP hydrogel retained a three-dimensional network structure and temperature-sensitive properties (Fig. 4E and F). The 3D network structure of the hydrogel was very important to sustain the release of bFGF from the bFGF-ASC-HP hydrogel. The *in vitro* release profiles of FITC-bFGF from both the HP hydrogel and the ASC-HP hydrogel indicate that the ASC-HP hydrogel had the slowest release rate. In addition to the 3D network structure of hydrogel, the following reasons contributed to the slow release by the bFGF-ASC-HP hydrogel. Firstly, a special interaction between the bFGF and the ASC scaffold, which was identified in the receptor staining experiment, hampered the diffusion of the bFGF into the hydrogel matrix. Secondly, we have already demonstrated that the HP hydrogel had an affinity for growth factor in a previous study, which also resulted in the sustained-release of bFGF. In an *i*n vitro cell proliferation study (Fig. 6), the bFGF-ASC–HP hydrogel was co-incubated with PC12 cells for 12 h, and the cell survival rate showed that the bFGF-ASC–HP hydrogel had good cytocompatibility with the PC12 cells, with no signs of cytotoxicity. Furthermore, less *in vivo* invasion of an ASC scaffold by immune cells than in an allologous spinal cord graft has been reported in the literature^19^.

Further, the efficiency and therapeutic effects of bFGF-ASC–HP hydrogel therapy have been extensively evaluated in SCI rats in this study. Astrocytes are a major component of a glial scar that forms subsequent to SCI that may prevent neuron regeneration. On the one hand, the bFGF-ASC–HP hydrogel significantly decreased the number of reactive astrocytes in SCI rats compared with the sham group, inhibiting the formation of a glial scar. On the other hand, the bFGF-ASC–HP hydrogel increased the expression of Nestin and GAP43, which could facilitate axonal growth and promote neural stem differentiation (Fig. 7). All these results together demonstrated that the bFGF-ASC–HP hydrogel increased neuronal survival and promoted axonal regeneration by reducing the glial scar.

SCI induces complex pathological processes and results in variable outcomes, which are influenced by many factors^58^,^59^. Apoptosis plays a vital role in the progressive degeneration of an injured spinal cord^3^. Caspase-3 immunohistochemical and TUNEL staining were used to evaluate apoptosis in each treatment group. Caspase-3 positive cells in the SCI group were significantly increased at 14 days after SCI. The number of positive Caspase-3 cells at 14 days in the bFGF-ASC–HP hydrogel group was significantly lower than in the other bFGF groups (*P* < 0.01). Furthermore, the TUNEL result was consistent with the Caspase-3 immunohistochemical staining result, which further indicated that the neuroprotective effect of the bFGF-ASC–HP hydrogel on SCI was related to the inhibition of apoptosis.

## Conclusions

In conclusion, ASC was used as a biologically active material to carry bFGF directly to an injured spinal cord. We utilized the advantages of bFGF, ASC and HP hydrogels to optimize the neuroprotection in SCI. Our study demonstrated that a bFGF-ASC-HP hydrogel significantly increased neuronal survival of and improved the functional recovery in acute SCI model rats. This study has provided extensive preclinical data that supports the use of this combined therapy to promote the application of natural and biological material in SCI.

## Additional Information

**How to cite this article**: Xu, H.-L. *et al*. Thermo-sensitive hydrogels combined with decellularised matrix deliver bFGF for the functional recovery of rats after a spinal cord injury. *Sci. Rep.* **6**, 38332; doi: 10.1038/srep38332 (2016).

**Publisher's note:** Springer Nature remains neutral with regard to jurisdictional claims in published maps and institutional affiliations.

## Supplementary Material

Supplementary Information
