# Supplementary material for: Thermo-sensitive hydrogels combined with decellularised matrix deliver bFGF for the functional recovery of rats after a spinal cord injury
Source: Sci Rep. 2016 Dec 6;6:38332. doi: 10.1038/srep38332 (PMC5138609; doi:10.1038/srep38332)
Supplement: Supplementary Information [file srep38332-s1.docx]

**Thermo-sensitive hydrogels combined with decellularised matrix deliver bFGF for the functional recovery of rats after a spinal cord injury**

He-Lin Xu^1a^, Fu-Rong Tian^1a^, Cui-Tao Lu^2a^, Jie Xu^1^, Zi-Liang Fan^1^, Jing-Jing Yang^1^ , Pian-Pian Chen^1^ , Ya-Dong Huang^3^, Jian Xiao^1*^ , Ying-Zheng Zhao^1,2**^

^1^ School of Pharmaceutical Sciences, Wenzhou Medical University, Wenzhou City, Zhejiang Province 325035, China.

^2^ The Second Affiliated Hospital of Wenzhou Medical University, Wenzhou, 325000, China;

^3^ Biopharmaceutical R&D Center of Jinan University, Guangzhou, Guangdong 510000, China

^*^ Correspondence to: J Xiao, School of Pharmaceutical Sciences, Wenzhou Medical University, Wenzhou City, Zhejiang Province 325035, China.. Email address: [2986069671@qq.com](mailto:2986069671@qq.com) (J Xiao)

^**^ Correspondence to: Y-Z Zhao, School of Pharmaceutical Sciences, Wenzhou Medical University, Wenzhou City, Zhejiang Province 325035, China. Email: [pharmtds@163.com](mailto:pharmtds@163.com) (Y-Z Zhao)

^a^ The first three authors contributed equally to this work.

**Supplementary Information**

Table S1. The daily bFGF percentage released from different formulations at fourth to seventh days (Mean±SD, n=3)

| Days | bFGF-ASC-HP | bFGF-HP | ASC+bFGF |
| --- | --- | --- | --- |
| D4 | 1.93±0.88% | 1.99±0.84% | 3.61±1.03% |
| D5 | 1.70±1.93% | 1.88±0.51% | 1.68±0.24% |
| D6 | 0.86±1.69% | 1.40±0.61% | 0.43±0.11% |
| D7 | 0.76±0.20% | 1.17±0.24% | 0.19±0.01% |

**ELISA assay and SDS-PAGE analysis of the remaining bFGF in different formulations**

In order to prove the stability of the remaining bFGF in different formulation after 7day of release, the amount of the remaining bFGF was further determined by ELISA and SDS-PAGE electrophoresis. After 7days of release, 10ml of PBS was added to the residue, fully mixed, and centrifuged to collect the supernatant. The supernatant was detected by ELISA kits according to instructions of reagent kits. Also, the residual bFGF was determined by SDS-PAGE electrophoresis analysis.

The remaining amount of bFGF in different formulations was summarized in Table S1. It was easily calculated that the bFGF percentage remained in different formulations was 24.16±0.25% for bFGF-ASC-HP, 19.35±0.35% for bFGF-HP and 8.11±0.23% for ASC-bFGF, respectively. These results were identical to the interfered value of the FITC-bFGF fluorescence in vitro release profile, indicating the remaining bFGF in each formulation is relatively stable. Also, SDS-PAGE analysis was used to ascertain whether the residual bFGF remained stable in the HP hydrogel and ASC-HP hydrogel. Results was shown in Figure S1, there was obvious strip of bFGF for the residual HP hydrogel and ASC-HP hydrogel, without appearance of additional strip, while residual bFGF-ASC exhibited a very faint strip because of very few residue of bFGF. These further indicated that the remaining bFGF was still stable in ASC-HP.

Table S2 The remaining bFGF amount determined by ELISA assay in different formulations after 7days of release (Mean±SD, n=3)

| Group | bFGF-ASC-HP | bFGF-HP | ASC+bFGF |
| --- | --- | --- | --- |
| (μg /ml) | 2.41±0.02 | 1.93±0.03 | 0.81±0.02 |


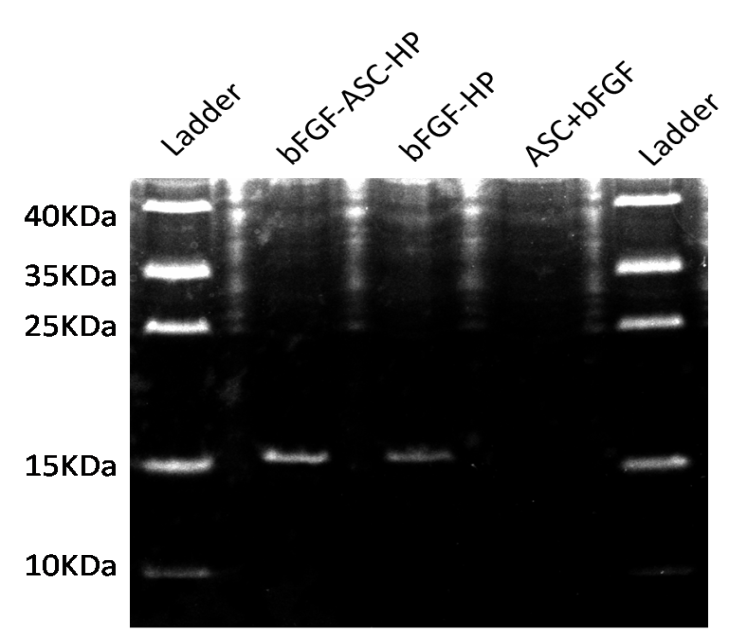


Fig.S1 SDS-PAGE graphs of the residual bFGF in different formulations
